# Suppression of Fluconazole Resistant *Candida albicans* Biofilm Formation and Filamentation by Methylindole Derivatives

**DOI:** 10.3389/fmicb.2018.02641

**Published:** 2018-11-06

**Authors:** Jin-Hyung Lee, Yong-Guy Kim, Vivek Kumar Gupta, Ranjith Kumar Manoharan, Jintae Lee

**Affiliations:** School of Chemical Engineering, Yeungnam University, Gyeongsan, South Korea

**Keywords:** methylindoles, *C. albicans*, biofilm, filamentation, *C. elegans*

## Abstract

*Candida albicans* is an opportunistic fungal pathogen and most prevalent species among clinical outbreaks. It causes a range of infections, including from mild mucosal infections to serious life-threatening candidemia and disseminated candidiasis. Multiple virulence factors account for the pathogenic nature of *C. albicans*, and its morphological transition from budding yeast to hyphal form and subsequent biofilm formation is regarded as the most important reason for the severity of *Candida* infections. To address the demanding need for novel antifungals, we investigated the anti-biofilm activities of various methylindoles against *C. albicans* using a crystal violet assay, and the metabolic activity was assessed by using a 2,3-bis (2-methoxy-4-nitro-5-sulfo-phenyl)-2H-tetrazolium-5-carboxanilide reduction assay. Changes in biofilm morphologies and thicknesses were determined by confocal laser scanning microscopy and scanning electron microscopy, respectively. Of the 21 methylindoles tested, 1-methylindole-2-carboxylic acid (1MI2CA) at 0.1 mM (17.5 μg ml^-1^) and 5-methylindole-2-carboxylic acid (5MI2CA) at 0.1 mM effectively inhibited biofilm formation by *C. albicans* DAY185 and ATCC10231 strains. Moreover, 1MI2CA and 5MI2CA both effectively inhibited hyphal formation, and thus, improved *C. albicans* infected nematode survival without inducing acute toxic effects. Furthermore, our *in silico* molecular modeling findings were in-line with *in vitro* observations. This study provides information useful for the development of novel strategies targeting candidiasis and biofilm-related infections.

## Introduction

Half million of people are afflicted by candidiasis worldwide, which has an annual mortality rate of 45–75% per year (Brown et al., 2012). Furthermore, candidiasis is regarded as the third-to-fourth most frequent nosocomial infection in the United States (Hajjeh et al., 2004; Wisplinghoff et al., 2004; Wenzel, 2007). *Candida albicans* is the main etiologic agent of candidiasis and causes both superficial and systemic infections in an expanding population of individuals with diminished host immunity (Sudbery, 2011). The Centre for Disease Control (CDC, United States) published a list of the top 18 drug resistant threats, which includes fluconazole resistant *C. albicans*, entailing urgent attention to prevent the spread of *Candida* infections (CDC, 2013).

The treatment of *C. albicans* is complicated because of its ability to proliferate as a biofilm (Gulati and Nobile, 2016). *C. albicans* colonizes host tissues, catheters, and indwelling medical devices and develops biofilms on biotic and abiotic surfaces (Nobile et al., 2006). *C. albicans* biofilms are composed of yeast and hyphal and pseudohyphal elements (Nobile et al., 2006). In particular, cells within biofilms are difficult to kill as they exhibit high levels of resistance to most clinically used antifungals. For example, MIC values are often more than 100-fold greater than those needed to eradicate free planktonic cells, and thus, biofilms serve as a reservoir for recurring fungal infections (Mathe and Van Dijck, 2013). The morphogenetic transition of *C. albicans* from yeast cells to the hypha form is crucial for robust biofilm formation, which represents the major virulence factor associated with serious *Candia* infections (Mayer et al., 2013). Also, various clinical isolates of *C. albicans* showed drug resistance against commercial antifungals, such as, azoles and polyenes (Taff et al., 2013). Currently, researchers are trying to develop new approaches to combat complicated *Candia* infections using novel antifungal drugs that target virulence mechanisms rather than growth inhibition, because the latter is associated with the emergence of multidrug resistance.

Indoles are intracellular signaling molecules produced by a variety of Gram-positive and Gram-negative bacteria (Lee and Lee, 2010). Several indoles and their derivatives have been reported to suppress the virulence and inhibit biofilm formation by several bacterial species, such as, *Staphylococcus aureus, Agrobacterium tumefaciens, Pseudomonas aeruginosa* (Lee et al., 2009, 2012, 2013, 2015, 2016), and *Vibrio cholera* (Mueller et al., 2009). Furthermore, some indole derivatives, such as, indole-3-acetonitrile, waikialoid A, shearinines, and 7-benzyloxyindole, have also been reported to inhibit *C. albicans* biofilm formation and hyphal development (Oh et al., 2012; Wang et al., 2012; You et al., 2013; Manoharan et al., 2018). While several natural indole derivatives showed antibiofilm activity, but the number of active compounds is limited and methylindoles have not investigated yet.

The goal of the present study was to identify methylindoles that potently inhibit biofilm formation and hyphal development by fluconazole-resistant *C. albicans*. The anti-biofilm properties of 21 methylindoles were investigated. Morphological characterizations and phenotypic switching of *C. albicans* cells were observed by confocal laser scanning microscopy and scanning electron microscopy, respectively. In addition, the most effective compounds were also evaluated with respect to hyphal inhibition and anti-biofilm efficacy in a *Caenorhabditis elegans* (a nematode) infection model.

## Materials and Methods

### Fungal Strains, Media, Methylindoles, and Growth Condition

The fluconazole resistant strain *C. albicans* DAY185 and ATCC10231 used in this study were obtained from Korean Culture Center of Microorganisms^1^. Both strains were preserved at -80°C in potato dextrose broth (PDB) medium supplemented with 30% glycerol until use. For repetitive culture, cells from glycerol stock were proliferated by streaking onto potato dextrose agar (PDA) plates and incubated for 48 h at 37°C. Also, yeast extract-peptone-dextrose (YPD) medium was used to confirm the results of PDB medium.

A loopful of cells were then inoculated into 250 ml Erlenmeyer flasks containing 25 ml of PDB medium and cultured in an orbital shaker at 250 rpm for 24 h at 37°C. All 21 methylindole derivatives (Supplementary Table S1) examined during the study were purchased from Sigma-Aldrich (St. Louis, MO, United States) and Combi Blocks Inc. (San Diego, CA, United States), and dissolved in dimethyl sulfoxide (DMSO); the concentration of DMSO in media did not exceed 0.1% (vol/vol) in any experiment. Cell growths and turbidities were measured using spectrophotometer (UV-160, Shimadzu, Japan) at 600 nm. Minimum inhibitory concentrations (MICs) were determined using the broth dilution method of the Clinical Laboratory Standards Institute (CLSI) with slight modification (Alastruey-Izquierdo et al., 2015), using 96-well polystyrene plates (SPL Life Sciences, South Korea). Cells were cultured overnight in PDB medium, diluted to a concentration of 10^5^ CFU/ml, added to wells in the absence or presence of varying concentrations (w/v) of methylindoles, and incubated for 24 h at 37°C. MIC was defined as the lowest concentration that inhibited microbial growth in PDB medium (300 μl) by at least 80% as assessed by spectrophotometry (620 nm) on 96-well polystyrene plates and colony counting. Each experiment was performed in triplicate.

### Screening of Methylindoles for Inhibition of *C. albicans* Biofilm Formation

*C. albicans* biofilms were developed on 96-well polystyrene plates (SPL Life Sciences, South Korea), as described previously (Lee et al., 2011; Manoharan et al., 2017b). Briefly, *C. albicans* cells were harvested from overnight cultures and added to PDB medium (300 μl) at 10^5^ CFU/ml and then cultured in the absence or presence of different concentrations of methylindoles for 24 h without shaking at 37°C. Biofilm formation was quantified by washing three times with water (to remove non-adherent cells), staining with crystal violet (0.1%) for 20 min, and then extracting the crystal violet with 95% ethanol. Absorbance was measured at 570 nm using a Multiskan EX microplate photometer (Thermo Fisher Scientific, Waltham, MA, United States) and results are presented as the averages of six replicates. Cell growth was measured at 620 nm.

### Biofilm Metabolic Activity – XTT Reduction Assay

A XTT [2,3-bis(2-methoxy-4-nitro-5-sulfophenyl)-2H-tetrazolium-5-carboxanilide sodium salt] (Sigma-Aldrich, St. Louis, MO, United States) reduction assay was used to quantify *C. albicans* metabolic activity in biofilms, as previously described (Ramage et al., 2001; Nett et al., 2011). A working solution of XTT and menadione solution was prepared at 20:1 (v/v) immediately prior to assays. Biofilms were produced as described above for the biofilm inhibition assay, by culturing the cells for 24 h without shaking at 37°C. Following biofilm formation, planktonic cells were aspirated and transferred to fresh 96-well polystyrene plates. Biofilms were then washed twice with sterile water to remove non-adherent cells. Phosphate buffer saline (PBS) was then added to XTT-menadione solution (3.76:1 v/v) and 200 μl of this mix was added to planktonic and prewashed biofilm cells, which were then incubated in the dark for 3 h at 37°C. The colored supernatant (100 μl) was carefully transferred to fresh microtiter plates, and absorbance was measured at 450 nm using a Multiskan EX microplate photometer (Thermo Fisher Scientific, Waltham, MA, United States).

### Colony Morphology of *C. albicans* on Solid Media

To investigate filament induction on solid media, *C. albicans* cells from glycerol stock, were plated on PDA agar plates including 10% fetal bovine serum supplemented with DMSO [less than 0.1% (vol/vol)] or methylindoles at 0.1 mM. Plates were then incubated for 6 days at 37°C, during which fungal colony morphologies were photographed after 2 days of interval using an iRiS^TM^ Digital Cell Imaging System (Logos Biosystems, South Korea).

### Confocal Laser Scanning Microscopy (CLSM)

*C. albicans* biofilms were developed on 96-well plates from re-suspended cells treated with methylindoles at 0.1 mM. After incubation for 24 h at 37°C, cells were washed with water three times to remove non-adherent cells and stained with carboxyfluorescein diacetate succinimidyl ester (Thermo Fisher Scientific, Invitrogen, MA, United States) (Weston and Parish, 1990). This method has been used previously (Nweze et al., 2012; Lee et al., 2018). Biofilms were visualized using an Ar laser (emission wavelength 500–550 nm) under a confocal laser microscope (Nikon Eclipse Ti, Tokyo) equipped with a 20× objective (Kim et al., 2012). Collected color confocal images (of at least 10 random positions) were constructed in three-dimensions using NIS-Elements C version 3.2 (Nikon eclipse). Assays were carried out using two independent cultures in triplicate. Biofilm formation was quantified by converting color confocal images (20 image stacks) to gray scale using ImageJ, and then using COMSTAT biofilm software (Heydorn et al., 2000) to calculate biomasses (μm^3^ μm^-2^), mean biofilm thicknesses (μm) and substratum coverages (%). Thresholding was fixed for all image stacks, and at least four positions and 20 planar images were analyzed per position.

### Scanning Electron Microscopy (SEM)

*C. albicans* hyphal formation was observed by SEM as described previously (Lee et al., 2014). Briefly, nylon membrane filters (GE Healthcare Life Sciences, United States) were cut into 0.5 cm × 0.5 cm pieces and placed in wells of 96-well polystyrene plates before *C. albicans* biofilm formation. Cells were inoculated into 200 μl of PDB medium at an initial turbidity of OD 0.05 at 600 nm in the presence or absence of methylindoles and cultured under static conditions for 24 h at 37°C. Samples were then rinsed with PBS and fixed in 2.5% (v/v) glutaraldehyde and formaldehyde for 24 h at 4°C, post-fixed with sodium phosphate buffer and osmium tetroxide overnight and dehydrated using an ethanol series (50, 70, 80, 90, 95, and 100%) and isoamyl acetate. The nylon filters were dried in a critical-point dryer and then mounted onto aluminum stubs and sputter-coated with platinum. Biofilm cells were examined under S-4200 scanning electron microscope (Hitachi, Japan) at 15 kV and magnifications ranging from ×600 to ×6,000.

### *Candida* Infection in the *Caenorhabditis elegans* Model

In order to investigate the effects of methylindoles on the virulence of *C. albicans, C. elegans* [*fer-15 (b26)*; *fem-1 (hc17)*] was infected with *C. albicans*, as previously described (Manoharan et al., 2017c). Briefly, a freshly prepared overnight culture of *C. albicans* (100 μl) was spread onto lawns on PDA plates and incubated for 48 h at 37°C to produce abundant cells and its virulent characteristics (Okoli et al., 2009). Synchronized adult worms were then allowed to feed on *C. albicans* lawns for 4 h at 25°C when worms were collected and washed three times with sterile M9 buffer. Approximately 10 worms were then pipetted into single wells of 96-well plates containing PDB medium and treated with solutions (300 μl) of the compounds investigated at final concentrations of 0.1 or 0.5 mM. Untreated cells were used as controls. Plates were then incubated at 25°C for 4 days without shaking. Three independent experiments were conducted in triplicate. Results are expressed as percentages of alive or dead worms, as determined by touching worms with a platinum wire after 4 days of incubation. Observations were made using an iRiS Digital Cell Imaging System (Logos Biosystems, Korea).

### Modeling and Docking Simulation

Molecular docking assays were performed, as previously described (Khan et al., 2017), to evaluate the interactions between methylindoles (1MI2CA and 5MI2CA) with the ATP binding sites of adenylyl cyclase (PDB, DOI: 10.2210/pdb1FX2/pdb) and compared with a known inhibitor of adenylate cyclase that is 2′,5′-dideoxyadenosine 3′-polyphosphate using Schrodinger software 11.4 (United States) (Rajasekharan et al., 2017). Higher negative Glide scores values indicate stronger binding interactions.

### Statistical Analysis

The statistical analysis was performed by one-way ANOVA followed by Dunnett’s test using SPSS version 23 (SPSS Inc., Chicago, IL, United States). *P*-values of <0.05 were regarded significant.

## Results

### Methylindole Derivatives Inhibited *C. albicans* Biofilm Formation

**FIGURE 1:**
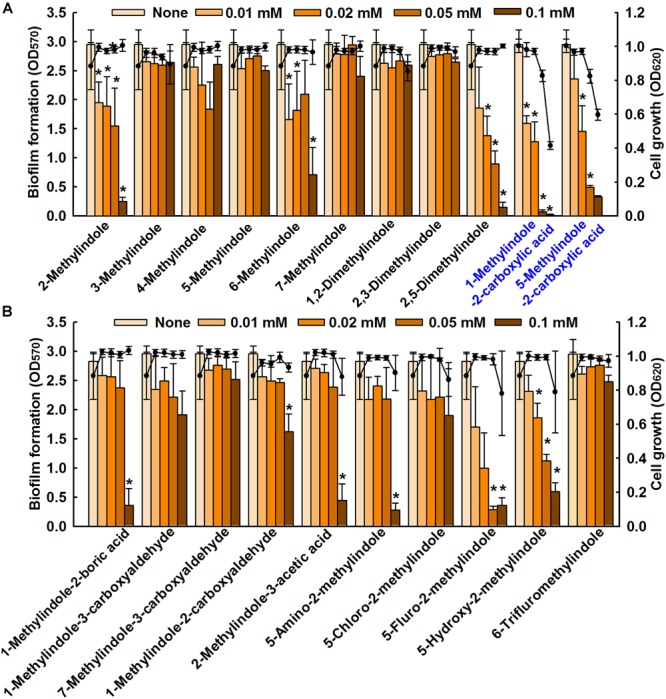
Inhibition of biofilm formation by methylindoles. The anti-biofilm activities of 21 methylindoles against *C. albicans* DAY185 were examined after incubation for 24 h **(A,B)**. The chemical structures of methylindoles are shown in Supplementary Table S1. At least two independent experiments were conducted (six wells per sample); error bars indicate standard deviations. ^∗^*p* < 0.05 vs. untreated controls. Bars indicate biofilm formation and lines indicate planktonic cell growth.

**FIGURE 2:**
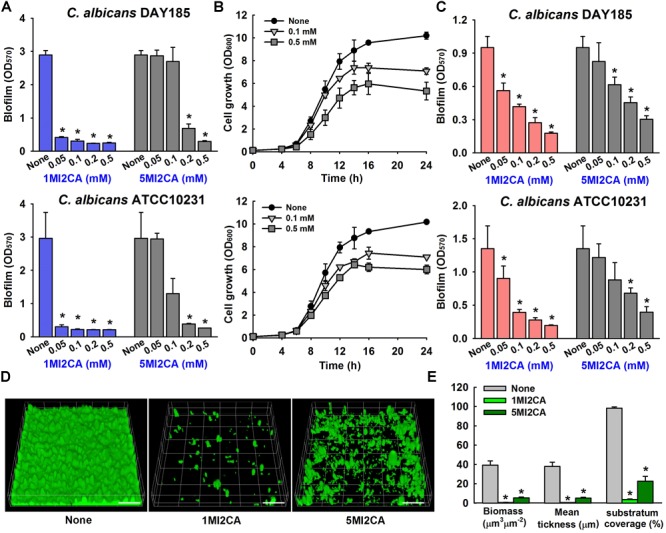
Effect of methylindoles on *C. albicans* biofilm formation and cell growth. The anti-biofilm activities of 1-methylindole-2-carboxylic acid (1MI2CA) and 5-methylindole-2-carboxylic acid (5MI2CA) were examined against *C. albicans* DAY185 and *C. albicans* ATCC10231 in PDB medium **(A)** and YPD medium **(C)** after incubation for 24 h. At least two independent experiments were conducted (six wells per sample). Planktonic cell growths of *C. albicans* DAY185 after incubation with 1MI2CA or 5MI2CA at their biofilm inhibitory concentrations of 0.1 and 0.5 mM for 24 h **(B)**. Confocal laser scanning microscopic observations of the effects of methylindoles on *C. albicans* biofilms **(D)**. Biofilm formation by *C. albicans* on polystyrene plates was observed in the presence of 1MI2CA or 5MI2CA at 0.1 mM by confocal laser microscopy. Scale bars represent 100 μm. Biofilm formation was quantified using COMSTAT **(E)**. Two independent experiments were conducted (six wells per sample). Error bars indicate standard deviations. ^∗^*p* < 0.05 vs. untreated controls.

We screened 21 commercially available methylindoles for their effects on biofilm formation by fluconazole resistant *C. albicans* DAY185 using a crystal violet assay. Initial screening showed that six of the 21 namely, 2-methylindole, 2,5-dimethylindole, 1-methylindole-2-carboxylic acid, 5-methylindole-2-carboxylic acid, 5-amino-2-methylindole, and 5-fluoro-2-methylindole at 0.1 mM significantly reduced *C. albicans* biofilm in PDB medium by more than 85% whereas other methylindole derivatives had less impact on *C. albicans* biofilm formation (Figures 1A,B). In particular, 1-methylindole-2-carboxylic acid (1MI2CA), and 5-methylindole-2-carboxylic acid (5MI2CA) at 0.1 mM inhibited biofilm formation by *C. albicans* DAY185 by 91 and 87%, respectively. In addition, at higher concentrations of 0.2 and 0.5 mM, 1MI2CA and 5MI2CA significantly and dose-dependently reduced biofilm formation by *C. albicans* DAY185 in PDB medium (Figure 2A). Similarly, the anti-biofilm activities of 1MI2CA and 5MI2CA were also observed in another strain of *C. albicans* ATCC10231 (Figure 2A). Specifically, 1MI2CA significantly reduced biofilm formation by *C. albicans* DAY185 by 92 and 96% at concentrations of 0.2 and 0.5 mM, respectively, and reduced biofilm formation by *C. albicans* ATCC10231 by 88, 93, and 95% at 0.1, 0.2, and 0.5 mM, respectively (Figure 2A). Both 1MI2CA and 5MI2CA slightly inhibited the planktonic cell growth of *C. albicans* DAY185 (by ∼30%) at a concentration of 0.1 mM (Figure 2B). The minimum inhibitory concentrations (MIC) of 1MI2CA and 5MI2CA against *C. albicans* DAY185 and ATCC10231 were ≥2 mM, while MICs of antifungal fluconazole are >10 mM (3060 μg/ml) in PDB and YPD media for the two strains. To confirm the antibiofilm activities in PDB medium, YPD medium was also used and found that 1MI2CA and 5MI2CA significantly inhibited biofilm formation by *C. albicans* DAY185 and ATCC10231 strains in a dose-dependent manner (Figure 2C). However, other active compounds 2-methylindole and 6-methylindole showed no antibiofilm activity on *C. albicans* ATCC10231 in PDB medium (data not shown). Hence, two most active compounds, 1MI2CA and 5MI2CA, were focused on further studies.

Confocal laser scanning microscopy (CLSM) images showed dense biofilm formation by untreated *C. albicans* cells, and remarkable reductions in biofilm adherence and thickness were observed in cells treated with 1MI2CA or 5MI2CA at 0.1 mM (Figure 2D). COMSTAT analysis showed that both 1MI2CA and 5MI2CA significantly reduced biofilm biomass (by 98.3 and 86.1%, respectively), average thickness (by 98.2 and 86.2%, respectively) and substrate coverage (by 96.2 and 76.9%, respectively) vs. untreated control cells (Figure 2E).

### Methylindoles Affected the Metabolic Activity of *C. albicans*

**FIGURE 3:**
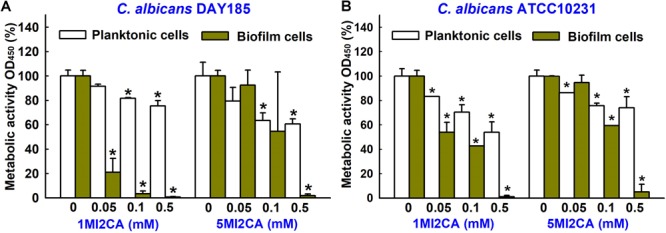
Effects of 1MI2CA and 5MI2CA on the metabolic activity of *C. albicans*. The metabolic activities of *C. albicans* DAY185 **(A)** and *C. albicans* ATCC10231 **(B)** planktonic cells and biofilms were quantified in the presence of methylindoles using an XTT reduction assay after incubation for 24 h. Results are presented as mean percentages of metabolic activity vs. untreated controls. Two independent experiments were conducted (six wells per sample); error bars indicate standard deviations. None indicates untreated samples. Error bars indicate standard deviations. ^∗^*p* < 0.05 vs. untreated controls.

XTT colorimetric assays were used to study the metabolic activities of fungal biofilm cells and planktonic cells in the presence or absence of 1MI2CA or 5MI2CA. Results are expressed as percentage metabolic activities of both treated and untreated cells. Treatment with 1MI2CA at 0.1 mM significantly reduced the metabolic activities *C. albicans* DAY185 and ATCC10231 biofilm cells by 97 and 58%, respectively, whereas 5MI2CA at 0.1 mM reduced metabolic activities by 66 and 41%, respectively. The viabilities of planktonic *C. albicans* DAY185 and ATCC10231 cells were slightly reduced (by ≤40%) by 1MI2CA or 5MI2CA at 0.1 mM (Figures 3A,B).

### Methylindoles Affected *C. albicans* Morphology and Hyphal Transition

**FIGURE 4:**
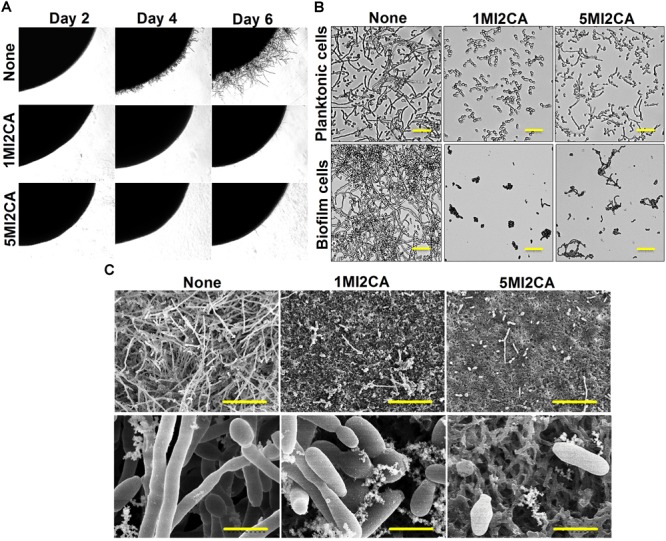
Effects of 1MI2CA and 5MI2CA on *C. albicans* DAY185 morphology and hyphal growth. *C. albicans* morphology on solid media **(A)**. *C. albicans* was streaked on PDA agar plates supplemented with 10% fetal bovine serum in the absence or presence of 1MI2CA or 5MI2CA (0.1 mM). Colony morphologies were photographed every 2 days at 37°C. Inhibition of the hyphal growths of planktonic and biofilm cells of *C. albicans* **(B)**. *C. albicans* was grown in PDB medium in the absence or presence of 1MI2CA or 5MI2CA (0.1 mM) at 37°C. Cultures were sampled after 24 h and photographed under a bright field microscope. The scale bar represents 100 μm. Microscopic observations (SEM) of the effects of the two methylindoles (0.1 mM) on biofilms and hyphal formation **(C)**. *C. albicans* cells were grown on nylon filter paper in the presence or absence of 1MI2CA or 5MI2CA and visualized by SEM. Scale bars represent 50 and 5 μm, respectively. At least two independent experiments were conducted. None indicates untreated control cells.

The effects of 1MI2CA or 5MI2CA on *C. albicans* morphology and hyphal growth were assessed by visual observation. Yeast cells were cultivated on PDA agar plates in presence or absence of 1MI2CA or 5MI2CA at 37°C. Initial filament formation was observed in untreated fungal colony after 4 days of incubation. 1MI2CA or 5MI2CA at 0.1 mM well inhibited filamentation until 6 days (Figure 4A).

The effects of 1MI2CA or 5MI2CA on *C. albicans* hyphal formation were examined in liquid medium (PDB) containing planktonic and biofilm cells under static anaerobic conditions at 37°C. In both independent experiments, untreated control cells showed massive hyphal formation after 24 h of incubation, but 1MI2CA (0.1 mM) treated cells showed no hyphal formation and cells remained in the rounded yeast form. Some cells (∼15%) treated with 5MI2CA at 0.1 mM developed hyphae (∼15%) (Figure 4B).

Scanning electron microscopy (SEM) showed 1MI2CA and 5MI2CA at 0.1 mM substantially reduced biofilm formation and suppressed hyphal transition on nylon membranes (Figure 4C). Untreated control biofilm cells exhibited large hyphae, pseudohyphae, and yeast cells, whereas methylindole treated cells showed greater numbers of yeast cells and fewer hyphae.

### Methylindoles Interacted With Adenylate Cyclase *in silico*

**FIGURE 5:**
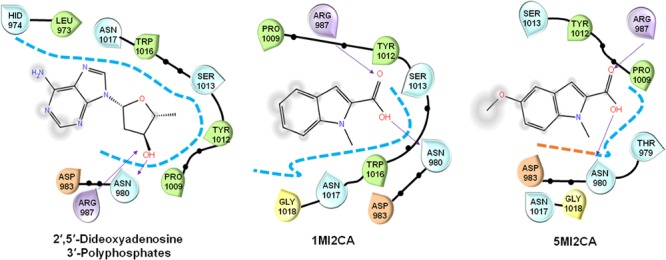
Molecular docking of the interaction between 1MI2CA or 5MI2CA and adenylate cyclase. The binding orientations of 1MI2CA and 5MI2CA within the active site of adenylate cyclase were similar to that of 2′,5′-dideoxyadenosine 3′-polyphosphate as a positive control.

Molecular docking was used to investigate the possible molecular mechanism of action of 1MI2CA and 5MI2CA. Adenylate cyclase of *C. albicans* plays a regulatory role in the cyclic AMP (cAMP) signaling pathway, which is essential for hyphal growth, virulence, and biofilm formation (Lu et al., 2014), and is thus viewed as a potential target for antifungal drug development. Anticipating that methylindoles might inhibit the function of adenylate cyclase, molecular docking was compared with 2′,5′-dideoxyadenosine 3′-polyphosphate (a known inhibitor of adenylate cyclase). As was expected, 2′,5′-dideoxyadenosine 3′-polyphosphate was found to form hydrogen bonds (binding energy: -4.09 kcal/mol) with catalytically important residues (arginine 987 and asparagine 980) of adenylate cyclase. Interestingly, both 1MI2CA and 5MI2CA formed hydrogen bonds with arginine 987 and asparagine 980 residues (Glide scores -3.90 and -4.07 kcal/mol, respectively) indicating interactions between both methylindoles (1MI2CA and 5MI2CA) and adenylate cyclase (Figure 5). However, the addition of cAMP up to 10 mM could not rescue the inhibitory effects of 1MI2CA and 5MI2CA (data not shown). Although speculative, 1MI2CA or 5MI2CA did not solely depend on the cAMP signaling pathway and could also affect other hyphae regulatory pathways.

### Methylindoles Suppressed the Virulence of *C. albicans* in the *Caenorhabditis elegans* Infection Model

**FIGURE 6:**
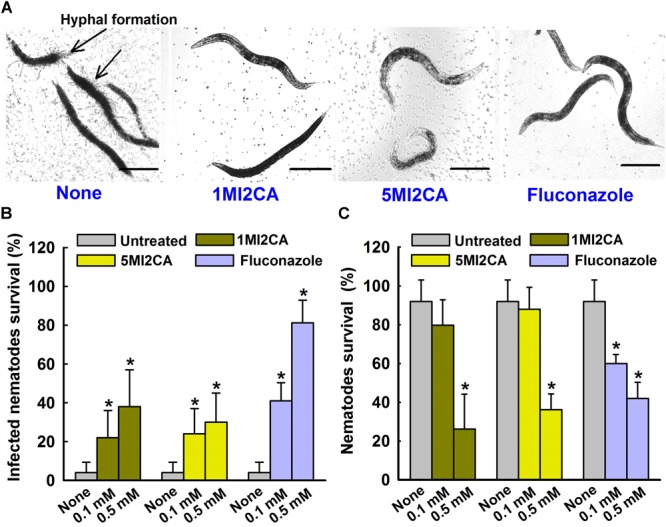
Effects of methylindoles on *C. albicans* virulence in the model of *C. elegans*. Microscopic images of *C. albicans* infected *C. elegans* in absence or presence of methylindoles (0.5 mM) or fluconazole as a positive control (0.1 mM) **(A)**. The arrows indicate hyphal formation in untreated infected nematodes and the scale bar represents 100 μm. The bar graph shows nematode percentage survival after exposure of *C. albicans* for 4 days to methylindoles (0.1 or 0. 5 mM) **(B)**. The toxicities of 1MI2CA and 5MI2CA were studied on non-infected nematodes by calculating survival rates after exposure for 4 days **(C)**. Fluconazole (0.1 or 0.5 mM) was used as the positive control. None indicates untreated controls. Worm survival was determined based on movement. At least two independent experiments were conducted. Error bars indicate standard deviations. ^∗^*p* < 0.05 vs. untreated controls.

We examined the effects of 1MI2CA and 5MI2CA on hyphal formation and virulence by *C. albicans in vivo* using a *C. elegans* nematode infection model. Visual microscopic observations showed the hyphal formation in untreated *C. albicans* infected nematodes (Figure 6A). However, no hyphal formation has been observed in nematodes treated with both 1MI2CA and 5MI2CA at 0.5 mM. Furthermore, microscopic observations of nematodes showed that *C. albicans* infection caused 96% fatality of nematodes in 4 days (Figure 6B). However, about 40% of nematodes survived after 4 days in the presence of 1MI2CA at 0.5 mM and >30% survived in the presence of 5MI2CA at the same concentration. Fluconazole (the positive control) at 0.1 mM had a worm survival rate of >80% at 4 days (Figure 6B). In addition to rescue treatment, the toxicities of 1MI2CA and 5MI2CA have also been investigated in *C. albicans* uninfected *C. elegans* model. The results showed that >80% of worms were survived at 0.1 mM concentration of both methylindoles (1MI2CA and 5MI2CA) whereas at 0.5 mM, it was found to be mild toxic titer (about 40% survival) for worms. Fluconazole was found to have similar chemical cytotoxicity effect (Figure 6C).

## Discussion

*C. albicans* biofilm infections are a serious problem for individuals with an implanted medical device, because they are resistant to the majority of clinical antifungal agents (Silva et al., 2017). Some indole derivatives like indole-3-acetonitrile, tetracyclic indoles, and 7-benzyloxyindole have been reported for their antivirulence effects and drug resistance reversal potential against *C. albicans* (Oh et al., 2012; Youngsaye et al., 2012; Manoharan et al., 2018), which prompted us to screen various commercially available methylindoles for their abilities to inhibit biofilm formation by fluconazole resistant *C. albicans* DAY185 (Figure 1). In the present investigation, we found two methylindoles, that is, 1MI2CA and 5MI2CA, significantly inhibited *C. albicans* DAY185 biofilm formation by >85% at 0.1 mM, which was 20-fold lower than its MIC for planktonic cells (Figure 2). These findings show the antibiofilm activity of two methylindoles was not due to fungicidal effects, which may reduce the chance of the development of drug resistance.

The presence of a carboxyl group in these methylindoles might importantly contribute to their anti-biofilm activities, possibly because of the moderate electron withdrawing ability of this group. It has been previously reported that the presence of an electron withdrawing group enhanced the antimicrobial effects of novel quinoline derivatives (Desai et al., 2017). In another report, carboxylic acid derivatives such as indole-2-carboxylic acid benzylidenehydrazides had stronger anticancer activities than other indole derivatives because they induced cancer cell apoptosis pathways (Zhang et al., 2004), and in a recent study, carboxamide derivatives of indole were found to exhibit better antifungal activities than propanamide derivatives (Olgen et al., 2008). Based on these reports, it would appear the enhanced antibiofilm activities of 1MI2CA and 5MI2CA are due to the presence of a carboxyl group (Figure 2).

The morphogenetic switch from the fungal yeast cells to the hyphal form is most investigated virulence attribute of *C. albicans* (Nobile and Mitchell, 2005; Lu et al., 2014), Notably, hyphae formation is mainly responsible for the adherence of mature fungal biofilm cells to host surfaces or implant medical devices (Almeida et al., 2008; Lu et al., 2014). Recently, attempts have been made to identify the antivirulence agents that specifically target hyphal transition in *C. albicans* (Li et al., 2015; Khan et al., 2017; Yun and Lee, 2017). In the present study, it was found that both methylindoles (1MI2CA and 5MI2CA) remarkably reduced hyphal formation (Figure 4). Collectively, these findings of visual microscopic observation and SEM analysis showed that both methylindoles reduce the virulence of *C. albicans* by suppressing filamentation and biofilm formation. Similar results have been reported for cedar leaf essential oil, emodin, and cinnamaldehyde immobilized gold nanoparticles against *C. albicans* (Janeczko et al., 2017; Manoharan et al., 2017c; Ramasamy et al., 2017). cAMP-dependent protein kinase A (cAMP-PKA) signaling with other factors causes yeast to hyphal transition in *C. albicans*, and adenylate cyclase (Cyr1) regulates cAMP function (Lu et al., 2014). Our docking results revealed possible molecular interactions between 1MI2CA and 5MI2CA and the ATP binding pocket of adenylate cyclase (Figure 5), indicating that both 1MI2CA and 5MI2CA may regulate the function of cAMP. Therefore, affecting the hyphal development is supported our *in vivo* findings that both methylindoles suppressed virulence by preventing hyphal formation (Figures 5, 6).

*Caenorhabditis elegans* infection models have proven to be useful for studies on the determinants of fungal virulence (Pukkila-Worley et al., 2009). We showed that the hyphal form of *C. albicans* rapidly kills *C. elegans* (Figure 6A). The yeast cells of *C. albicans* are ingested into the digestive tract of *C. elegans* and where it undergoes the morphological transition that is hyphal form. Such hyphal cells persistently penetrate the collagenous cuticle layer of the worm, which results the death of the worm. On the other hand, in the presence of 1MI2CA or 5MI2CA (at 0.1 mM and 0.5 mM, respectively), *C. elegans* survival was markedly increased (Figure 6B), which we believe it was due to the inhibition of hyphal formation. Therefore, it could be concluded that the hyphal formation is critical for *C. albicans* pathogenesis in *C. elegans* infection model. Furthermore, this result is in accord with earlier reports on magnolol, honokiol, linalool α-longipinene (Sun et al., 2015; Manoharan et al., 2017a). We also examined the toxicities of 1MI2CA and 5MI2CA uninfected *C. elegans* (Figure 6C). It was found neither methylindole had a significant toxic effect and that both were effectively non-toxic at their biofilm inhibitory concentration of 0.1 mM.

## Conclusion

In the present study, two methylindoles (1MI2CA and 5MI2CA) diminished the virulence of *C. albicans* by reducing hyphal development and biofilm formation *in vitro* and *in vivo*, which suggests both of these methylindoles have potential therapeutic applications against biofilm-associated *C. albicans* infections. In future, *in vivo* efficacy and pharmacokinetic studies using murine models would enable the establishment of the half-life of the molecule, bioavailability, and cytotoxicity.

## Author Contributions

J-HL, Y-GK, VG, RM, and JL performed the experiments, analyzed the data, and wrote the manuscript. J-HL and JL designed the study. All authors have read and approved the final manuscript.

## Conflict of Interest Statement

The authors declare that the research was conducted in the absence of any commercial or financial relationships that could be construed as a potential conflict of interest.

## Footnotes

1 http://www.kccm.or.kr/
