# Supplementary material for: Suppression of Fluconazole Resistant Candida albicans Biofilm Formation and Filamentation by Methylindole Derivatives
Source: Front Microbiol. 2018 Nov 6;9:2641. doi: 10.3389/fmicb.2018.02641 (PMC6232606; doi:10.3389/fmicb.2018.02641)
Supplement: Supplementary file 1 [file Table_1.docx]

**Supplementary Table S1. Chemical structures of 21 methylindoles examined.**

| **Methylindoles** | **Structure** | **Methylindoles** | **Structure** |
| --- | --- | --- | --- |
| **2-Methylindole** | [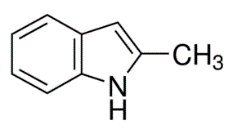](https://www.sigmaaldrich.com/catalog/product/aldrich/m51407?lang=ko&region=KR) | **1-Methylindole-2-boric acid MIDA ester** | [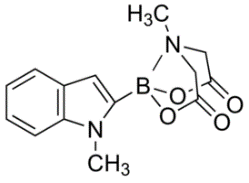](https://www.sigmaaldrich.com/catalog/product/aldrich/758299?lang=ko&region=KR) |
| **3-Methylindole** | [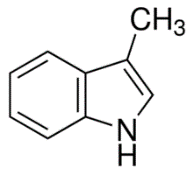](https://www.google.com/url?sa=i&source=imgres&cd=&cad=rja&uact=8&ved=2ahUKEwiMrJzivqLbAhVHxLwKHX8jCPUQjRx6BAgBEAU&url=https://www.sigmaaldrich.com/catalog/product/aldrich/w301912?lang%3Den%26region%3DUS&psig=AOvVaw3pozdzyyy80J3skpSQhyZG&ust=1527393618720810) | **1-Methylindole-3-corboxyaldehyde** | [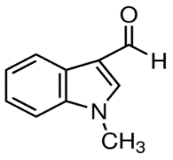](https://www.sigmaaldrich.com/catalog/product/aldrich/357987?lang=ko&region=KR) |
| **4-Methylindole** | [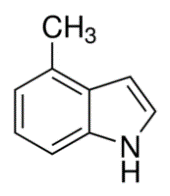](https://www.sigmaaldrich.com/catalog/product/aldrich/246301?lang=ko&region=KR) | **7-Methylindole-3-corboxyaldehyde** | [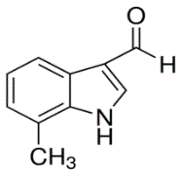](https://www.sigmaaldrich.com/catalog/product/aldrich/m2502?lang=ko&region=KR) |
| **5-Methylindole** | [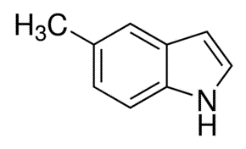](https://www.sigmaaldrich.com/catalog/product/aldrich/222410?lang=ko&region=KR) | **1-Methylindole-2-carboxyaldehyde** | 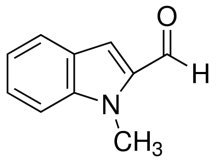 |
| **6-Methylindole** | [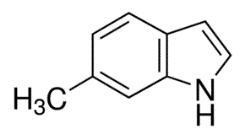](https://www.sigmaaldrich.com/catalog/product/aldrich/246328?lang=ko&region=KR&gclid=Cj0KCQjw6J7YBRC4ARIsAJMXXsdjkwRKKr2s7xRucFnaPPmt6D0w_8SgUveOJGgptbxMMoPjNjYfQE4aAsYPEALw_wcB) | **2-Methylindole-3-acetic acid** | 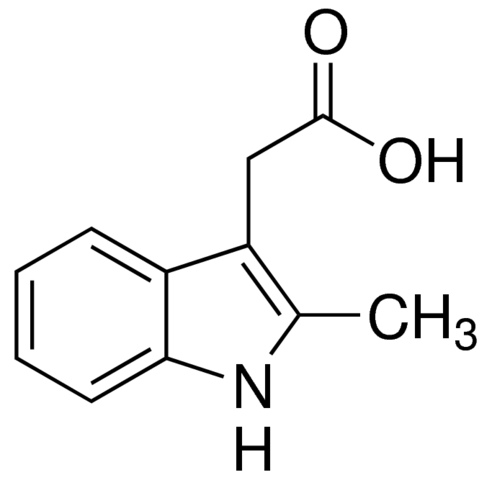 |
| **7-Methylindole** | [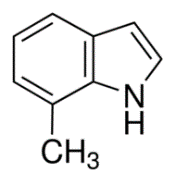](https://www.sigmaaldrich.com/catalog/product/aldrich/m51490?lang=ko&region=KR) | **5-Amino-2-methylindole** | [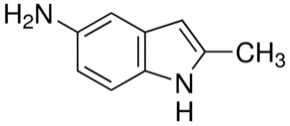](https://www.sigmaaldrich.com/catalog/product/aldrich/525588?lang=ko&region=KR) |
| **1,2-Dimethylindole** | [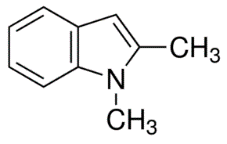](https://www.sigmaaldrich.com/catalog/product/aldrich/d165603?lang=ko&region=KR) | **5-Chloro-2-methylindole** | [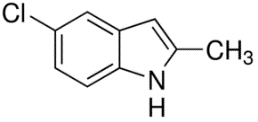](https://www.sigmaaldrich.com/catalog/product/sial/c52802?lang=ko&region=KR) |
| **2, 3-Dimethylindole** | [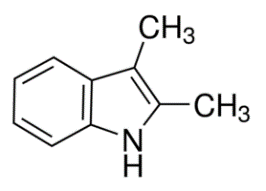](https://www.sigmaaldrich.com/catalog/product/aldrich/120812?lang=ko&region=KR) | **5-Fluro-2-methylindole** | [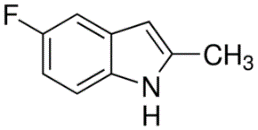](https://www.sigmaaldrich.com/catalog/product/aldrich/511536?lang=ko&region=KR) |
| **2, 5-Dimethylindole** | [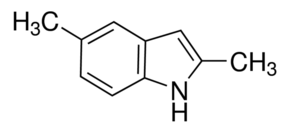](https://www.sigmaaldrich.com/catalog/product/aldrich/d166006?lang=ko&region=KR) | **5-Hydroxy-2-methylindole** | 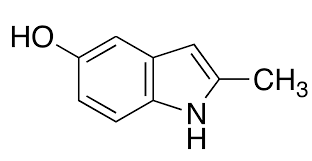 |
| **1-Methylindole-2-carboxylic acid** | [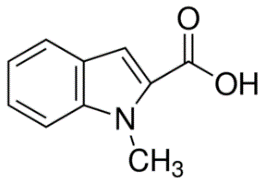](https://www.sigmaaldrich.com/catalog/product/aldrich/134155?lang=ko&region=KR) | **6-Trifluromethylindole** | [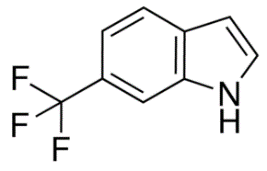](https://www.sigmaaldrich.com/catalog/product/aldrich/cds001039?lang=ko&region=KR) |
| **5-Methylindole-2-carboxylic acid** | [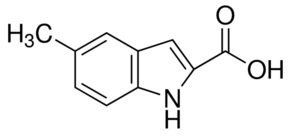](https://www.google.com/url?sa=i&source=images&cd=&cad=rja&uact=8&ved=2ahUKEwiskOKnl7HbAhXGe7wKHfs6DHAQjRx6BAgBEAU&url=https://www.sigmaaldrich.com/catalog/product/aldrich/m2627?lang%3Den%26region%3DUS&psig=AOvVaw1wYo8WYbTLNjqe6EgGrVlX&ust=1527898409359115) |  |  |
